# Key genomic regions identified through selection signatures distinguish cattle breeds reared in Sardinia Island

**DOI:** 10.1186/s12864-025-12204-6

**Published:** 2025-11-05

**Authors:** Maria Chiara Fabbri, Guido Gomez Proto, Francesco Tiezzi, Simone Callegaro, Francesco Sirtori, Alessandro Crovetti, Riccardo Bozzi

**Affiliations:** 1 Department of Agriculture, Food, Environment, and Forestry (DAGRI), University of Florence, Piazzale delle Cascine 18, Florence, 50144 Italy; 2 Department of Agronomy, Food, Natural Resources, Animals and Environment (DAFNAE), University of Padova, Viale dell’Università 16, Legnaro, 35020 Italy

**Keywords:** Homozygosity, Selective sweeps, Population structure, Local breeds, Haplotypes

## Abstract

**Background:**

Natural and artificial selection are believed to have left traces on the genomes of livestock, often characterized by the fixation of variants linked to traits under selection. These footprints differentiate populations genetically. The selection signatures of four Italian island cattle breeds — Sarda, Sardo Bruna, Sardo Modicana, and Limousine — were studied here using Integrated Haplotype Score, Cross-population Extended Haplotype Homozygosity, and Fixation Index analysis.

**Results:**

Several signals of positive selection have been identified in the four beef cattle breeds, with many overlapping regions (31.6% of genes found on recurrent haplotypes). The cross-population extended haplotype homozygosity analysis revealed three notable regions recurring in pairwise comparisons: from 36,681,743 to 38,001,622 bp on BTA6, containing 66 SNPs; from 66,502,254 to 70,354,943 bp on BTA11 with 42 markers; and from 22,781,305 to 24,452,175 bp on BTA14 with 26 SNPs. In total, 134 SNPs were identified as potentially useful discriminant variables across the four breeds. More precisely, SAM and SAR differentiated from the other breeds for the selection signature on BTA6,while SAB for that on BTA11, and finally LIM presented a breed-specific region on BTA14.

The region on BTA 6 appears to be the most promising for the genes located there (e.g., *LAP3*, *MED28 *, *FAM184B*, *DCAF16*, *NCAPG*, and *LCORL*), which have been identified in multiple studies as candidate genes for body and bone weight, as well as growth development.

**Conclusions:**

This comprehensive genomic analysis of selection signatures in four beef cattle breeds reared within the same geographic area reveals distinct patterns shaped by both local adaptation and long-term selection pressures. Shared selective sweeps involving key growth and development genes underscore common functional targets across breeds, while unique breed-specific regions highlight their genetic distinctiveness and adaptive strategies. This study could lay the foundation for future valorization and traceability strategies that will be cheap for farmers' and breeders’ associations.

**Supplementary Information:**

The online version contains supplementary material available at 10.1186/s12864-025-12204-6.

## Background

Selection signatures are genomic patterns that indicate past or ongoing selection [1]. These signatures often show a local reduction in genetic variability both upstream and downstream of the advantageous mutation, which quickly becomes fixed in the population over several generations. Identifying selection signatures has become a key focus in livestock genetics because it helps uncover genes and beneficial mutations that provide a selective advantage. Additionally, it offers insights into evolutionary history and, importantly, aids in the characterization and differentiation of various breeds.

Several approaches have been proposed to detect selection signatures, as described by Saravanan et al. (2020) [1]. These include methods based on linkage disequilibrium (LD), allele frequency spectrum, and reduced local variability. These methods can be classified into two groups: within-population and between-population statistics. The within-population statistics investigate footprints of selection by comparing genomic data within populations. In contrast, between-population statistics evaluate the degree of differentiation due to locus-specific allele frequencies between different populations. Within-population statistics can be further differentiated between LD test-related and reduced variability investigation [1]. The most widely used approach of the latter method is the Runs of Homozygosity (ROH) analysis while the integrated haplotype score (iHS) is the most widespread statistic for LD tests. It measures how unusual haplotypes are around a SNP compared to the whole genome, incorporating the recombination distance into the equation, which was elaborated by Voight et al. (2006) [2]. The iHS is an extension of the EHH (extended haplotype homozygosity) value described by Sabeti et al. (2002) [3], which represents the probability that a pair of chromosomes carries homozygous core haplotypes. From the EHH is derived the rEHH (relative extended haplotype homozygosity), which is calculated to compare the EHH values of two haplotype blocks: high rEHH values and high frequency in the population mean that the haplotype is under positive and recent selection [3].

Haplotype information is also used in between-population analysis. One of the most recent methods in this context is the cross-population extended haplotype homozygosity (XP-EHH) [4]. To calculate XP-EHH between populations A and B, IHH values (integrated haplotype homozygosity, i.e., the integration of EHH of the entire sample in the population) for each population are calculated separately. The positive and negative XP-EHH scores suggest that the selection occurred in populations A and B, respectively.

In this study, the two methods described above have been used and applied to three local breeds and one cosmopolitan breed widely reared in Italy. Italy is rich in livestock genetic diversity, although the top five cattle populations reared are Holstein (*n* = ~ 2,302,000), crossbreds (*n* = ~ 1,377,000), Piemontese (*n* = ~ 318,000), Limousine (*n* = ~ 295,000), and Charolaise (*n* = ~ 201,300) (Sistema Informativo Veterinario – BDN, https://www.vetinfo.it/j6_statistiche/index.html#/). These breeds are classified as cosmopolitan, and the selected breeds (excluding animals registered as “crossbred”) are regularly enrolled in official Herd Books. A peculiar situation is Sardinia Island, where the crossbred population counted ~ 120,000 individuals, followed by Sardo Bruna (*n* = 56,880, a local Sardinian breed), Holstein, Limousine, and another local breed (Sarda) at the 5th position in the ranking of the most reared breeds with approximately 20,000 animals (Sistema Informativo Veterinario – BDN, https://www.vetinfo.it/j6_statistiche/index.html#/). Crossbreds are economically important for the meat production industry, but mustn’t be included as purebreds in the official registers. This situation arises from breed management (e.g., extensive rearing, which may lead to possible ancestry assignment errors) and economic incentives: Sardinian farmers received a monetary prize for each animal reared in pure breeding. This prize applied when both sire and dam were registered, although a genomic test was mandatory for the offspring. Given these assumptions, developing a low-cost and time-efficient method to genetically differentiate these breeds is crucial.

This study aims to detect selection signatures and their ability to differentiate among the four beef breeds (Sarda, Sardo Bruna, Sardo Modicana, and Limousine) primarily raised in Sardinia, and often admixed with each other. The island’s traditional management practices, including natural insemination in multi-bred herds, have limited gene flow from mainland Italy and other countries. However, this type of management increases the possibility of finding admixed animals, a negative aspect in terms of meat traceability and valorization. In addition, these conditions complicate the reconstruction of population history and increase the time required for such analysis.

## Methods

### Animals, genotyping, and quality control

The study included a total of 11,859 genotyped animals. The local breeds consisted of 3,531 individuals, namely 1,031, 2,161, and 339 animals for Sarda (SAR), Sardo Bruna (SAB), and Sardo Modicana (SAM), respectively. In addition, Limousine (LIM) amounted to 8,348 individuals.

All individuals were genotyped with the GeneSeek GGP-LDv4 33 k (Illumina Inc., San Diego, California, USA), which includes 30,111 SNP markers. The SNP map positions were updated based on the ARS-UCD2.0 bovine genome assembly using BovineMine v1.6. Genotype phasing was performed with Beagle software v.5.4 [5], using default software parameters (i.e., burn-in = 3, iterations = 12). Subsequently, data quality control was performed, and only SNPs located on the 29 autosomes, with less than 10% missing values and a minor allele frequency (MAF) greater than 1%, were retained for the following analyses. The MAF threshold was arbitrary chosen based on similar studies [6, 7] and a previous investigation on the same breeds [8], which finally identifies the 1% as the best value to use (It excludes not only monomorphic SNPs, but also rare variants and potential genotyping errors).

### Population structure analysis

Principal components analysis (PCA) was used to investigate the data structure of distinct populations and performed using the flag --*pca* in PLINK v1.9 [9]. More precisely, PCA produces orthogonal projections of the original data (i.e. SNPs retained after the quality control described above), highlighting how different populations are structured (between and within groups). Individuals that did not plot (visually) clearly within one of the four breeds’ clusters were considered potential crossbreds and consequently removed from selection signature analyses. A simple visual approach has been chosen to give an immediate and clear output, also easily repeatable by the Breeder Association under farmers request Two Principal Component Analyses were performed: one including only the Sardinian breeds and the other including the Limousine breed as well.

### Selection signature analyses

Haplotypes were constructed with PLINK2 software [9] using --*export haps flag*.

After removing potential crossbreds identified from PCA, within-population signatures of selection were computed using the iHS [2], calculated for each autosomal SNP through the rehh R package [10]. Large positive or negative iHS values indicate unusually long haplotypes carrying the ancestral or derived alleles, respectively. Candidate regions were identified using a scan window of 500 kb with no overlap, with a minimum number of extremal markers equal to 5, and a threshold MAF of 5%.

To identify selection signatures between populations, SAR was compared to each Sardinian population using XP-EHH (Cross-population extended haplotype homozygosity) [5], and the same method was applied to compare each local breed to LIM. As a result, five pairwise XP-EHH analyses were performed (namely, SAR vs. SAB, SAR vs. SAM, LIM vs. SAB, LIM vs. SAM, LIM vs. SAR). The XP-EHH scores were calculated with the rehh R package using default settings [11]. The purpose of calculating XP-EHH is to detect alleles with increased frequency, approaching fixation or near-fixation in the reference breed, compared to the other breeds analyzed. In essence, XP-EHH tests whether a specific genomic site is homozygous in one population but still polymorphic in another by comparing the EHH scores of two populations.

In these pairwise analyses, the SAR first and the LIM after were considered as reference populations. Therefore, only the highly positive XP-EHH scores identified SNPs under selection in the reference population but not in the other breeds. The significance threshold selected for all these analyses (within and between selection signature methods) was set at the 99.9th percentile of the empirical parameter distribution.

Linkage disequilibrium (LD) was investigated between the pairwise SNPs within the significant regions indicated by the highly positive XP-EHH scores. LD was measured using the r^2^, which is the squared correlation of the alleles at two loci, obtained with the flag --ld-window-r2 0 in PLINK software [9].

The fixation index (F_ST_) was used as a measure of genetic differentiation between populations. F_ST_ quantifies differences in allele frequencies between populations, with values ranging from zero (no differentiation) to one (complete differentiation, where subpopulations are fixed for different alleles). F_ST_ values are useful for identifying signatures of selection between groups, that is, loci where alleles are fixed differently across populations, thus revealing how divergent selection can shape genomic patterns. Negative F_ST_ values, which lack biological interpretation, were set to zero [11]. Loci were plotted relative to their physical position within each autosome. SNPs exceeding the 99.9th percentile of the empirical parameter’s distribution were considered outliers.

### Annotation of candidate genome regions

Genes located within ± 500 kbp from the top 0.01% iHS signals, the top 0.01% F_ST_ values, and genes within the significantly extended stretches of homozygosity which differentiated breeds (i.e., within XP-EHH) were retrieved from the Bos taurus genome assembly ARS-UCD2.0 (https://www.ncbi.nlm.nih.gov/datasets/genome/GCF_002263795.3/) using the biomart R package [12]. Identification of potential candidate genes for selection was obtained by comparing our results with those in the literature.

## Results

### Animals, genotyping, and quality control

After applying quality control, no animals were excluded. Consequently, 11,859 animals were included in this study, while the number of SNPs that passed the quality control varied among breeds (Table 1).

**Table 1:** Number of SNPs and animals after data quality control

Breed	*n*. SNPs	*n*. Animals
LIM	23,691	8,328
SAB	24,261	2,161
SAM	26,211	339
SAR	25,432	1,031

*LIM* Limousine, *SAB* Sardo Bruna, *SAM* Sardo Modicana, *SAR* Sarda

### Population structure analysis

**Fig. 1:**
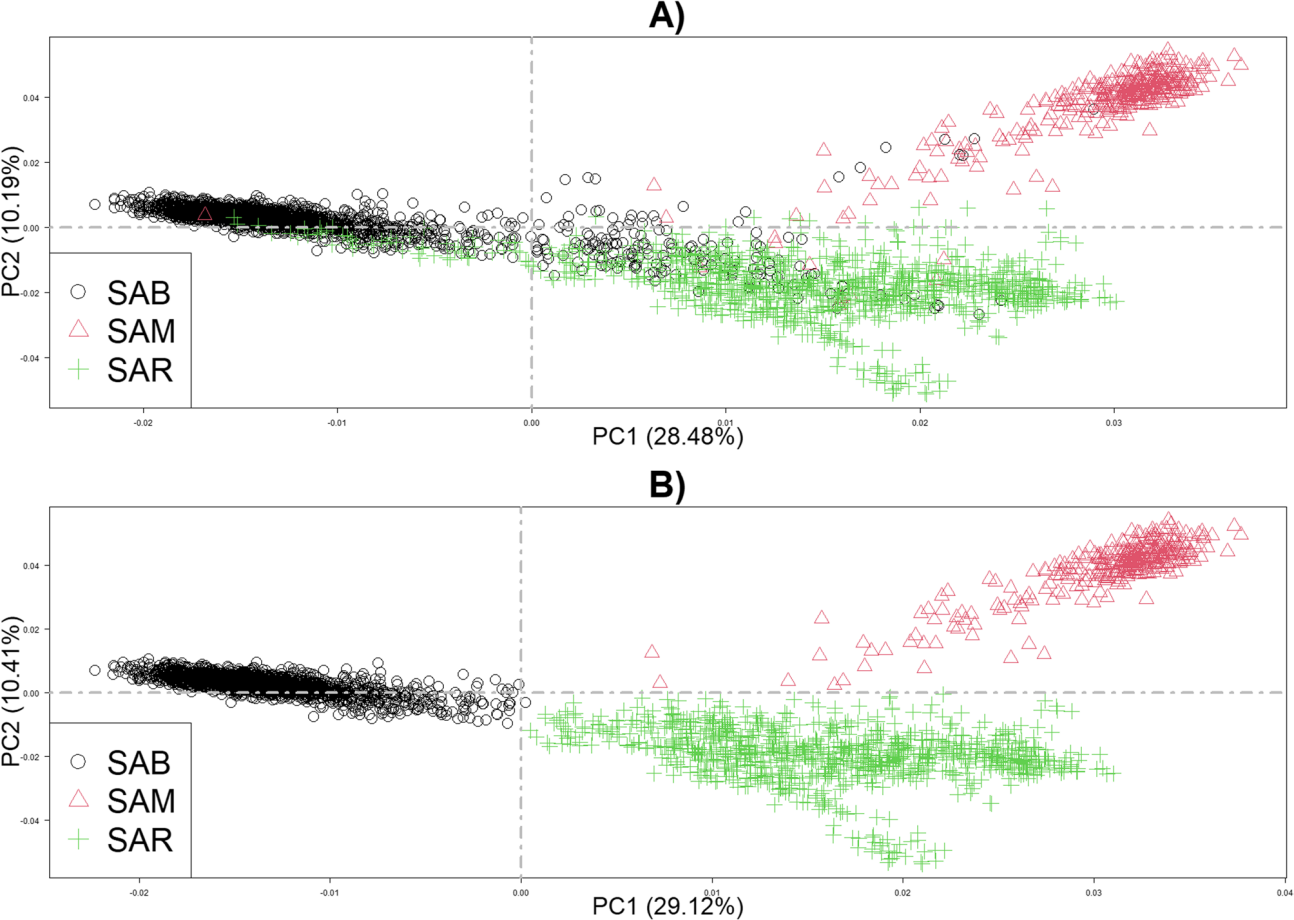
PCA of Sardinian Cattle Breeds: (**A**) using the whole dataset; (**B**) after removing potential crossbreds. PCA = Principal Components Analysis, SAB = Sardo Bruna, SAM = Sardo Modicana, SAR = Sarda

Multiple Principal Component Analyses were performed. Firstly, only Sardinian breeds were plotted to verify the clusters; indeed, including the cosmopolitan breed with a different history, origin, and management could decrease the variability between local groups. As shown in Fig. 1A, some individuals (*n* = 253) appeared to be admixed. These animals were removed from the dataset, and PCA was newly conducted (Fig. 1B). Three well-defined clusters were found, with the lowest within-population variability in SAB. Instead, SAR showed high variability within the breed, with slight evidence of population stratification. Consequently, these remaining individuals were used in the PCA, where LIM was also included.

**Fig. 2:**
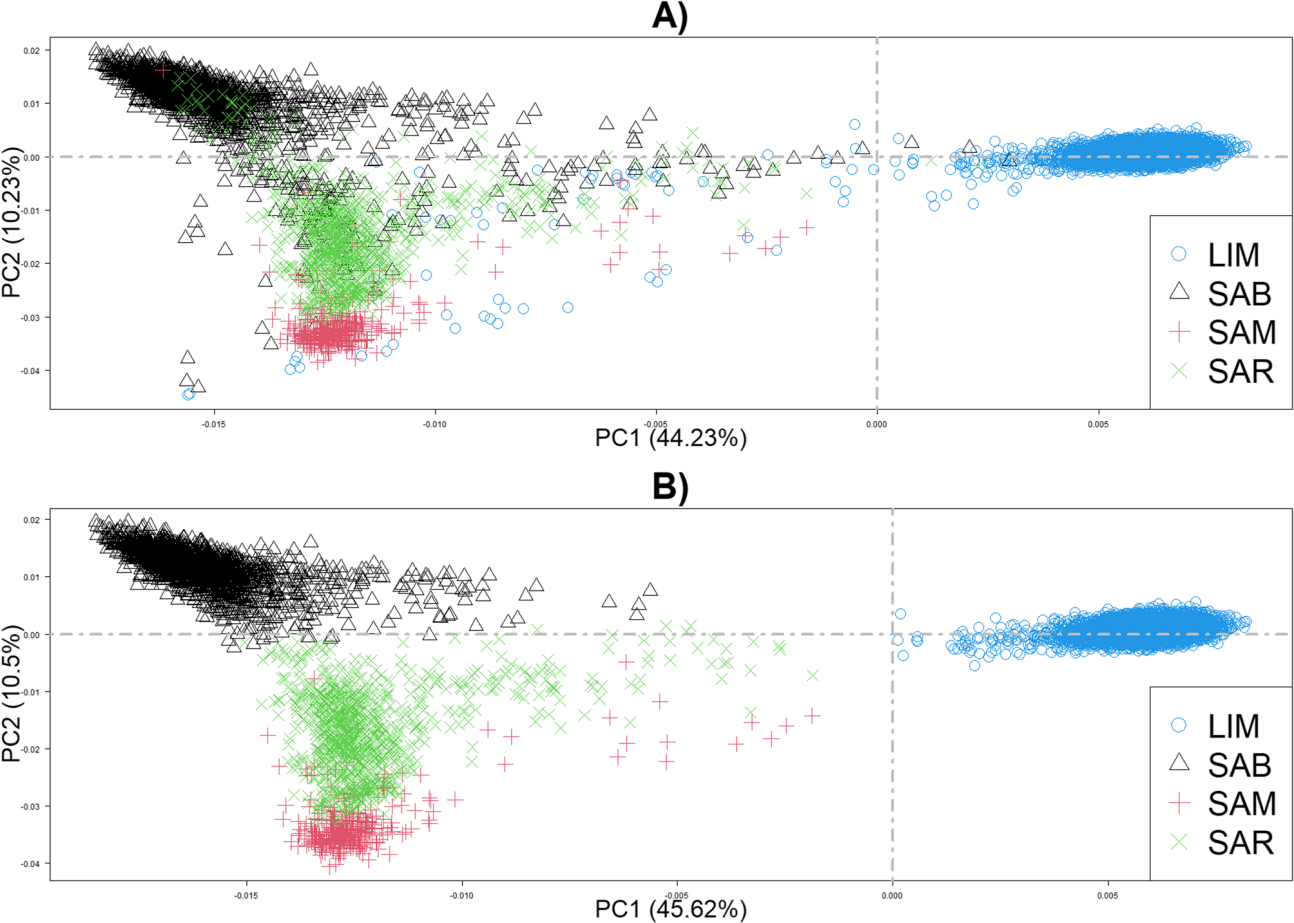
PCA of Sardinian and Limousine: **A**) using the whole dataset; **B**) after removing the potential crossbreds. PCA = Principal Components Analysis, LIM = Limousine, SAB = Sardo Bruna, SAM = Sardo Modicana, SAR = Sarda

Figure 2 shows that the Sardinian animals clustered closer, as expected, when LIM is included, plotting separately. However, several potential crosses were also identified in LIM (Fig. 2A) and then excluded from the dataset. Figure 2B illustrates the genetic similarities based on SNP data among the 4 breeds included for signature selection analyses.

After the removal of potential admixed animals, the final number of individuals for LIM was 8,266, while for the three Sardinian breeds it was 963, 1,986, and 329 in SAR, SAB, and SAM, respectively (a total of 11,544 individuals).

### Selection signature analyses

**Fig. 3:**
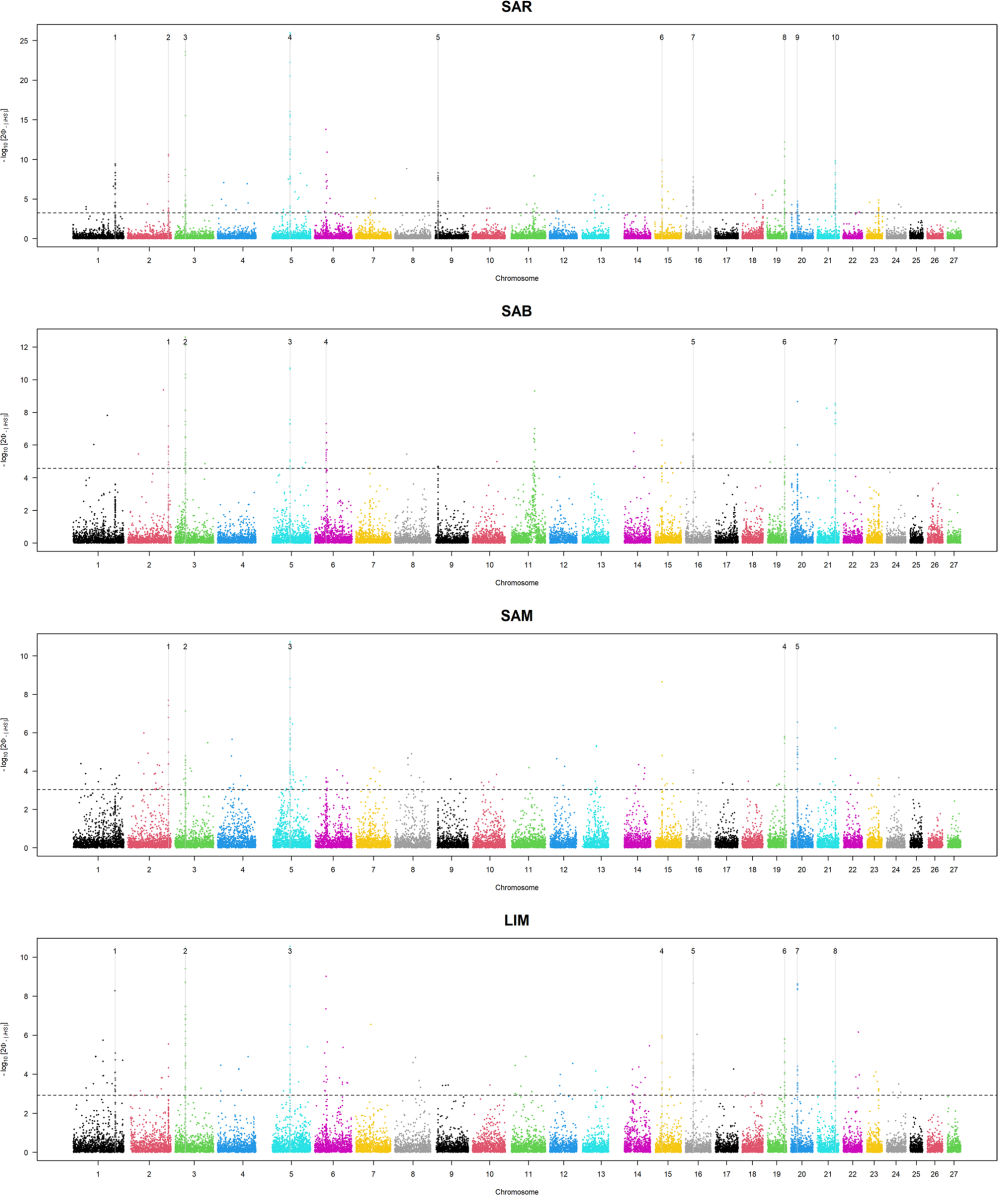
Manhattan plot of iHS signals for the four cattle beef breeds. LIM= Limousine, SAB = Sardo Bruna, SAM = Sardo Modicana, and SAR = Sarda

We initially performed an iHS analysis to detect signatures of positive selection within each cattle breed. The threshold corresponding to the top 0.01% |iHS| values led to numerous potential candidate regions in each cattle breed, as shown in Fig. 3. The top 0.01% |iHS| values resulted in 3.03, 3.26, 4.57, and 2.92 for SAM, SAR, SAB, and LIM, respectively. To ensure a more stringent evaluation, the genomic regions were considered only if they contained at least five extreme markers (i.e., SNPs with an |iHS| value exceeding the previously defined threshold). For this reason, some peaks in Fig. 3 were not highlighted or considered in the subsequent analyses. SAR showed the highest number of significant iHS signals, i.e., 10, while SAM was the breed with the lowest number of significant peaks revealed (*n* = 5).

Detailed information on putative signatures of selection for each breed was provided in Table 2. A considerable number of extreme markers were observed in certain genomic regions. The term “extreme” was used to define SNPs by having an iHS score above the specified threshold. Notably, LIM exhibited three regions with a high number of extreme markers, located on BTA3, BTA16, and BTA20. For SAB, the corresponding regions were identified on BTA3 and BTA16, while in SAR, significant IHS signals were observed on BTA1, BTA3, BTA5, BTA16, and BTA21. SAM showed two regions of high SNP density located on BTA5 and BTA21.

**Table 2:** Details of selection signatures using the |iHS| score identified in each breed

	BTA	START (bp)	END (bp)	*N*. markers	*N*. extreme markers
LIM	1	131,000,000	131,500,000	60	14
	3	32,500,000	33,500,000	128	27
	5	56,000,000	56,500,000	44	14
	15	23,500,000	24,000,000	81	9
	16	24,500,000	25,500,000	91	29
	19	56,000,000	56,500,000	50	11
	20	22,000,000	22,500,000	116	26
	21	57,000,000	57,500,000	68	10
SAB	2	127,000,000	127,500,000	38	5
	3	32,500,000	33,500,000	106	23
	5	56,000,000	56,500,000	43	8
	6	37,000,000	37,500,000	15	10
	16	25,000,000	25,500,000	63	19
	19	56,000,000	56,500,000	49	6
	21	57,000,000	57,500,000	70	10
SAR	1	131,000,000	132,000,000	79	27
	2	126,500,000	127,000,000	39	8
	3	32,500,000	33,500,000	128	21
	5	55,500,000	56,500,000	78	36
	9	10,000,000	11,000,000	82	11
	15	23,500,000	24,000,000	79	14
	16	25,000,000	25,500,000	65	23
	19	56,000,000	56,500,000	51	18
	20	22,000,000	22,500,000	113	10
	21	56,500,000	57,500,000	95	28
SAM	2	127,000,000	127,500,000	40	5
	3	32,500,000	33,000,000	77	9
	5	55,500,000	56,500,000	82	38
	19	56,000,000	56,500,000	49	5
	20	22,000,000	22,500,000	116	17

*BTA* Bos taurus autosome, *LIM * Limousine, *SAB* Sardo Bruna, *SAM* Sardo Modicana, and *SAR* Sarda

**Fig. 4:**
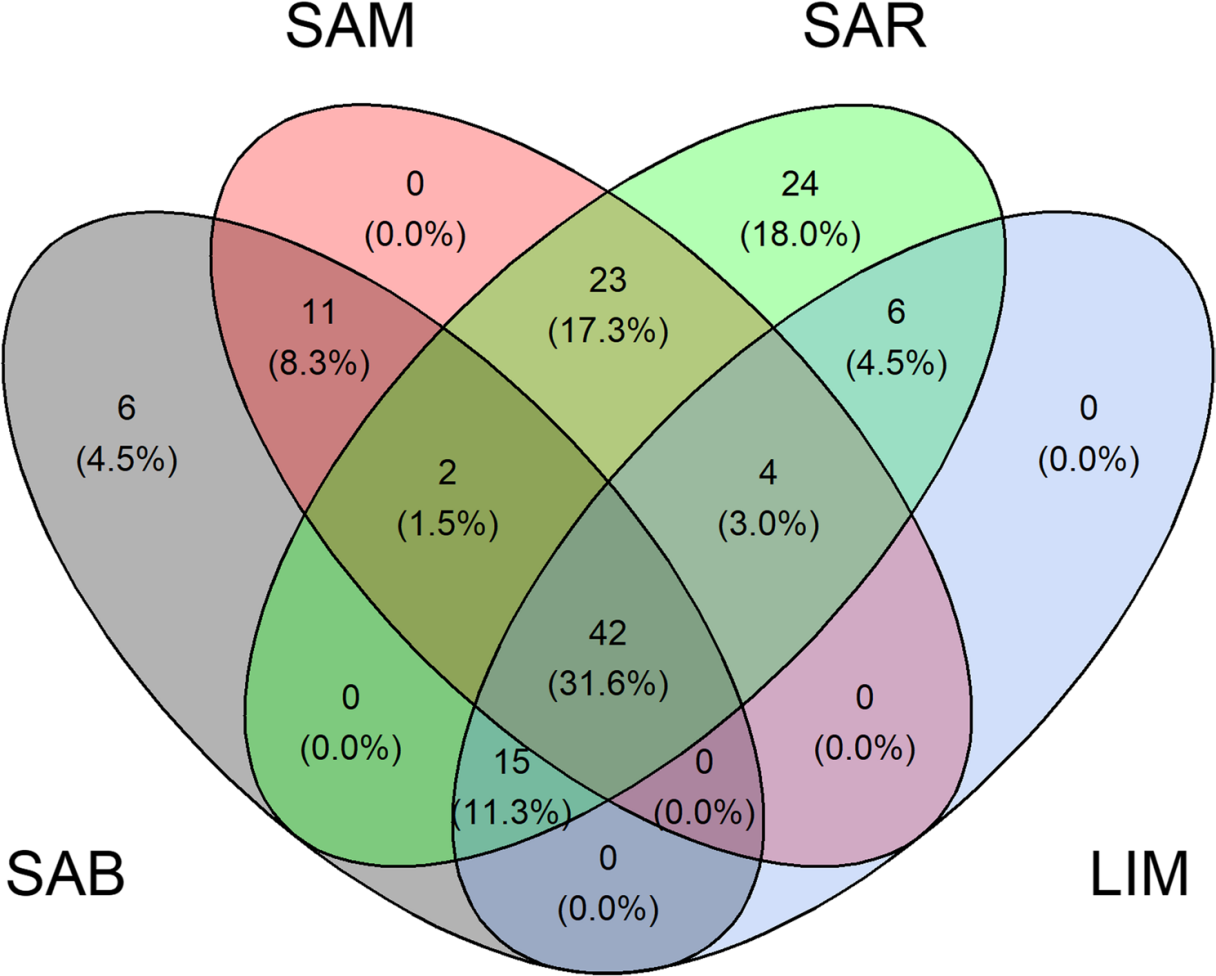
Venn diagram of genes located ±500 kbp from iHS signals. LIM = Limousine, SAB = Sardo Bruna, SAM = Sardo Modicana, and SAR = Sarda

Several candidate regions of selection overlapped across the four breeds. Genes located within ± 500 kbp segments of significant iHS signals were identified and compared to explore common or breed-specific genes. Figure 4 shows that 31.6% of genes were mapped to overlapping significant regions among the four breeds (Additional File 1); 20 genes were located on BTA19, 16 on BTA5, and 6 on BTA3. SAB and SAR revealed regions that were not significant in the other breeds (4.5% − 6 genes and 18% − 24 genes, respectively). The specific genes found in the unique segments in SAB included *LAP3* (Leucine Aminopeptidase 3), *MED28* (Methionine Sulfoxide Reductase B3), *FAM184B* (Family With Sequence Similarity 184 Member B), *DCAF16* (DDB1 And CUL4 Associated Factor 16), *NCAPG* (Non-SMC Condensin I Complex Subunit G), and *LCORL* (Ligand Dependent Nuclear Receptor Corepressor Like). The 24 specific genes identified in SAR selection signatures were: *RPS6KA1* (Ribosomal Protein S6 Kinase A1), *HMGN2* (High Mobility Group Nucleosomal Binding Domain 2), *DHDDS* (Dehydrodolichyl Diphosphate Synthase Subunit), *LIN28A* (Lin-28 Homolog A), *ZNF683* (Zinc Finger Protein 683), *CRYBG2* (Crystallin Beta-Gamma Domain Containing 2), *CD52* (CD52 Molecule), *UBXN11* (UBX Domain Protein 11), *SH3BGRL3* (SH3 Domain Binding Glutamate Rich Protein Like 3), *CEP85* (Centrosomal Protein 85), *CATSPER4* (Cation Channel Sperm Associated 4), *CNKSR1* (Connector Enhancer Of Kinase Suppressor Of Ras 1), *FAM110D* (Family With Sequence Similarity 110 Member D), *ZNF593* (Zinc Finger Protein 593), *C1orf232*, *PDIK1L* (PDLIM1 Interacting Kinase 1 Like), *NDUFB1* (NADH: Ubiquinone Oxidoreductase Subunit B1), *CPSF2* (Cleavage And Polyadenylation Specific Factor 2), *TRIP11* (Thyroid Hormone Receptor Interactor 11), *ATXN3* (Ataxin 3), *TC2N* (Tandem C2 Domains, Nuclear), *FBLN5* (Fibulin 5), *B3GAT2* (Beta-1,3-Glucuronyltransferase 2), and *OGFRL1* (Opioid Growth Factor Receptor Like 1).

**Fig. 5:**
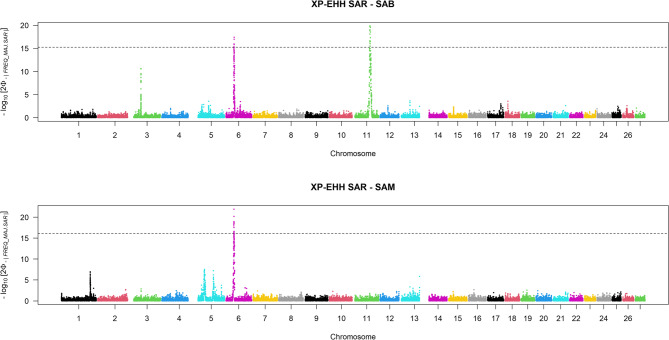
Manhattan plots of selection signatures by comparing the Sardinian cattle breeds. SAB = Sardo Bruna, SAM = Sardo Modicana, SAR= Sarda

The second Selection Signatures Analysis consisted of estimating the cross-population extended haplotype homozygosity as an indicator of positive selection (XP-EHH). Firstly, XP-EHH was evaluated in SAB and SAM compared to SAR, which should be the original breed from which SAB and SAM crossed. The same threshold of the top 0.01% of XP-EHH scores showed differences in cross-population extended haplotype homozygosity among Sardinian breeds (Fig. 5). Specifically, SAR appeared to differ from SAM and SAB in the regions located on BTA6 (36,681,743–38,001,622 bp and 37,142,174–38,001,622 bp, respectively) and BTA11 (66,502,254–69,619,152 bp). Additional File 2 provides support for this differentiation, indicating that the negative XP-EHH values observed in SAR, compared to SAB and SAM, reflect a positive selection signature in the latter. The haplotype encompassing these regions was thus identified as a candidate under selection in both populations.

**Fig. 6:**
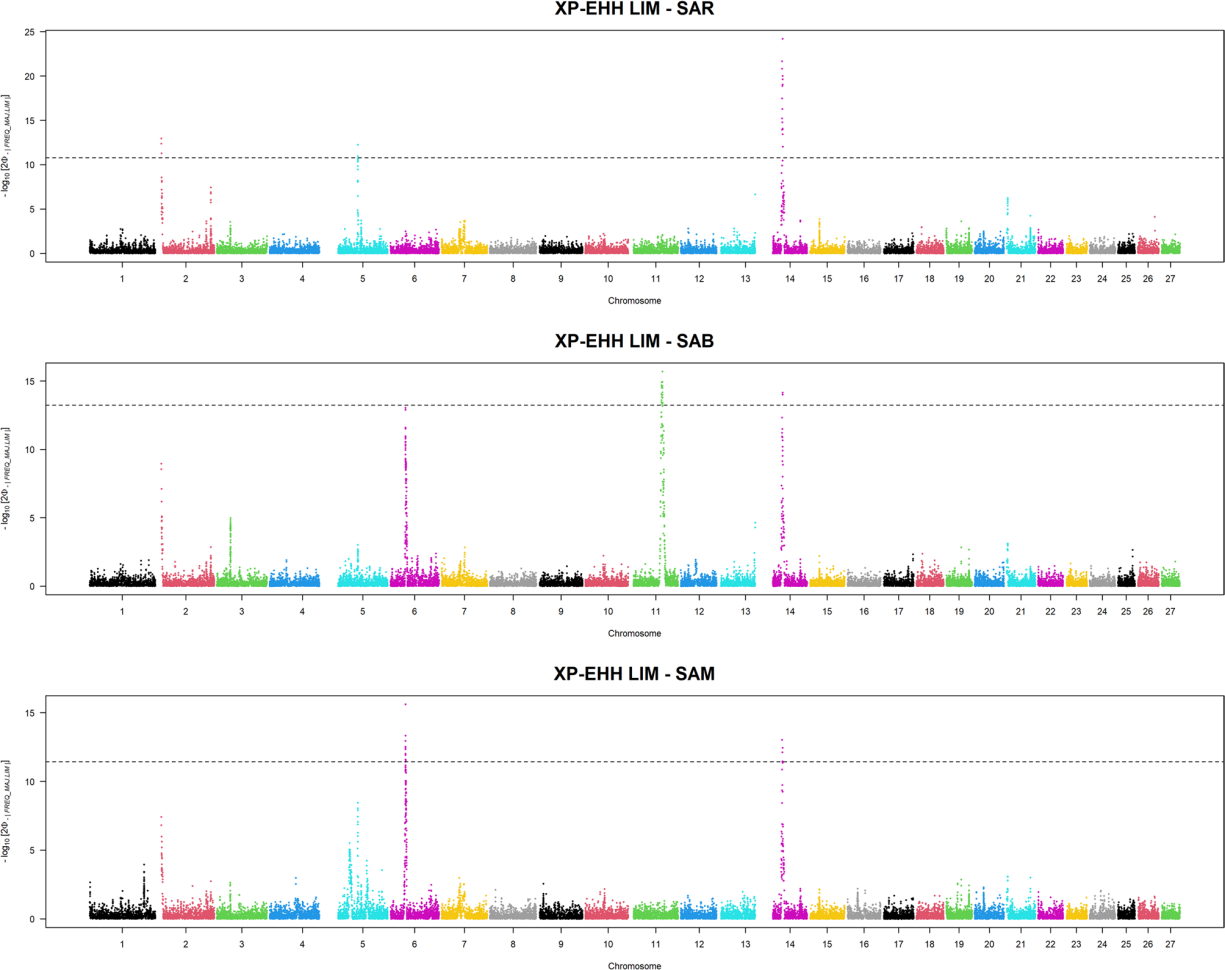
Manhattan plots of selection signatures by comparing the Sardinian cattle breeds with the cosmopolitan Limousine. LIM = Limousine, SAB = Sardo Bruna, SAM = Sardo Modicana and SAR= Sarda

Lastly, pairwise comparisons were also performed considering the LIM breed (Fig. 6), and the putative selection signatures identified in each Sardinian breed were evaluated. These comparisons revealed candidate regions on BTA6 for SAM. In contrast, the LIM vs. SAB comparison highlighted a region on BTA14 for LIM, which may represent a strong signal of selection in the cosmopolitan breed (Additional File 2).

Table 3 provides detailed information on the physical positions of the detected signatures of selections that show the most extreme |XP-EHH| scores.

**Table 3:** Genomic regions associated with the top 0.1% of |XP-EHH| and the SNPs under positive selection

Breeds	BTA	Start (bp)	End (bp)	*n*.SNPs
SAR vs. SAM	6	36,681,743	38,001,622	26
SAR vs. SAB	6	37,142,174	38,001,622	15
SAR vs. SAB	11	66,502,254	69,619,152	17
LIM vs. SAR	2	9,068,816	9,262,784	3
LIM vs. SAR	5	48,229,383	48,333,093	2
LIM vs. SAR	14	22,781,305	24,452,175	17
LIM vs. SAM	6	36,850,859	37,335,860	18
LIM vs. SAM	14	22,982,433	23,749,112	5
LIM vs. SAB	6	36,857,461	36,857,461	1
LIM vs. SAB	11	66,857,203	70,354,943	21
LIM vs. SAB	14	23,737,521	23,749,112	2

*|XP-EHH|* cross-population extended haplotype homozygosity scores, *BTA* Bos taurus autosome, *LIM* Limousine, *SAB* Sardo Bruna, *SAM* Sardo Modicana, and *SAR* Sarda

One region on BTA2 and one on BTA5 were revealed, but only in the LIM vs. SAR pairwise comparison. Three recurrent regions were identified and subsequently examined in depth (i) from 36.7 Mb to 38 Mb on BTA 6 containing 56 and from those, 15 and 26 SNPs seemed to be under selection in SAM and SAB compared to SAR; (ii) from 66.5 Mb to 70 Mb on BTA 11, where 81 markers are located and from those 17 and 21 are under selection in SAB compared to SAR and LIM, respectively; and (iii) from 22.7 Mb to 24 Mb on BTA 14 harboring 26 SNPs and from those 17, 5 and 4 markers are under selection in LIM compared to SAR, SAM and SAB respectively.

Linkage Disequilibrium has been investigated among the SNPs within the blocks described above, and the pairwise values of *r*^*2*^ are presented in Additional File 3 (Figure S1). The average pairwise *r*^*2*^ for the 66 SNPs in the BTA6 region was 0.24, while it reached 0.7 and 0.14 in the BTA11 and BTA14 regions (42 and 26 SNPs, respectively). These results demonstrated that the selection signatures identified were not only influenced by LD block structure artefacts.

Genes within the early referenced blocks were reported in Table 4. No genes were found within the segment on BTA2, while only *MSRB3* was detected in the BTA5 region under positive selection in LIM. The genomic region richest in genes was that on BTA11 (32 genes), identified as a potential selection signature in SAB, followed by the segment on BTA14 (11 genes), clearly under selection in the LIM breed. Nine genes were identified in the significant BTA6 segment, revealing a positive signature of selection in SAB and SAM.

**Table 4:** Genes within the genomic regions associated with the top 0.1% of |XP-EHH| scores

BTA	Start-End position (bp)	Gene name
2	9,068,816-9,262,784	none
5	48,229,383 − 48,333,093	*MSRB3*
6	36,681,743 − 38,001,622	*SPP1*,* MEPE*,* IBSP*,* LAP3*,* MED28*,* FAM184B*,* DCAF16*,* NCAPG*,* LCORL*
11	66,502,254 − 69,619,152	*C1D*,* DNAAF10*,* PNO1*,* PPP3R1*,* CNRIP1*,* PLEK*,* FBXO48*,* APLF*,* PROKR1*,* ARHGAP25*,* BMP10*,* GKN2*,* GKN1*,* ANTXR1*,* GFPT1*,* NFU1*,* AAK1*,* ANXA4*,* GMCL1*,* MXD1*,* ASPRV1*,* PCBP1*,* C11H2orf42*,* TIA1*,* PCYOX1*,* SNRPG*,* EHD3*,* CAPN14*,* GALNT14*,* CAPN13*,* LCLAT1*,* LBH*
14	22,781,305 − 24,452,175	*XKR4*,* TMEM68*,* TGS1*,* LYN*,* RPS20*,* MOS*,* PLAG1*,* CHCHD7*,* SDR16C5*,* SDR16C6*,* PENK*,* BPNT2*

*|XP-EHH|* cross-population extended haplotype homozygosity scores, *BTA* Bos taurus autosome

**Fig. 7:**
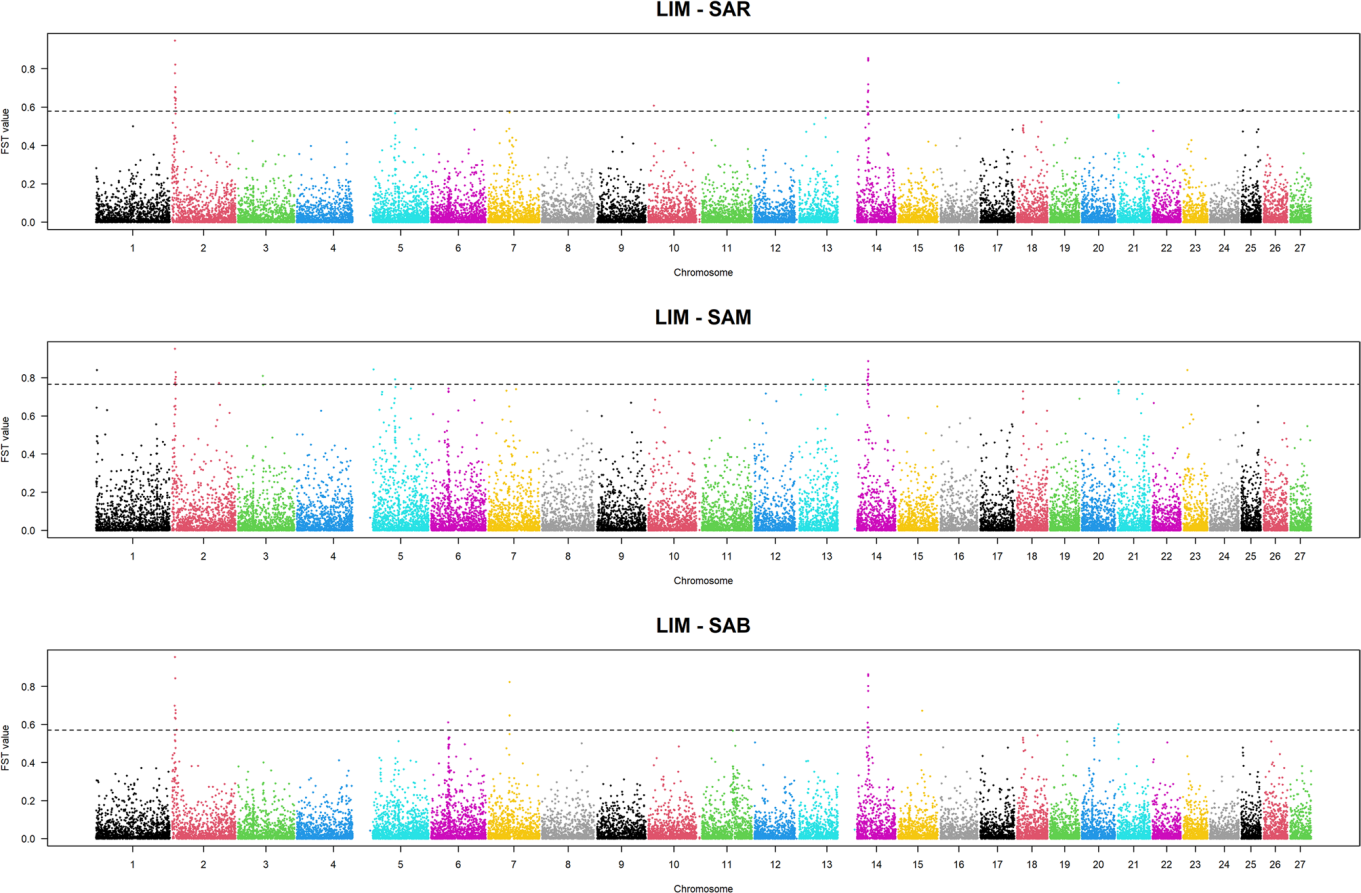
Manhattan plots of F_ST_ values by comparing the Sardinian cattle breeds with the cosmopolitan Limousine. LIM = Limousine, SAB = Sardo Bruna, SAM = Sardo Modicana and SAR= Sarda

F_ST_ values were calculated by comparing the cosmopolitan breed (LIM) against each of the three Sardinian populations. The top 0.1% F_ST_ thresholds were 0.57 for LIM vs. SAR and vs. SAB, and 0.76 for LIM vs. SAM. The Manhattan plots in Fig. 7 display the genomic distribution of F_ST_ values across autosomes.

Notably, pronounced peaks were observed on BTA2 and BTA14 in all pairwise comparisons, suggesting population differentiation at these loci. The list of genes identified with the F_ST_ approach is reported in the Additional File 4. In total, 156 genes were located within genomic windows exceeding the respective F_ST_ thresholds. Among them, 22 genes overlapped with genomic regions identified by the XP-EHH method (Table 4). The genes were distributed across BTA14, BTA6, and BTA5, including functionally relevant loci such as *PLAG1* (PLAG1 Zinc Finger), *SPP1* (Secreted Phosphoprotein 1), and *MSRB3*. The remaining 134 genes were uniquely identified by F_ST_. Of these, 48 were located on chromosomes BTA2, BTA5, and BTA14, already highlighted by XP-EHH, although they were situated upstream and downstream of the XP-EHH-defined regions. Specifically, 26 genes were mapped on BTA2 between 5.15 Mb and 8.87 Mb, from 9.83 to 10.7 Mb, and around 100 Mb, flanking the XP-EHH signals. On BTA14, additional genes were located outside but close to the XP-EHH interval, and on BTA5, six genes were situated upstream and downstream of *MSRB3.* Additionally, a region on BTA6 containing genes not detected by XP-EHH was located approximately 20 kb from the top-ranked region containing *SPP1*.

## Discussion

Traditional breeding practices in Sardinia, shaped by the island’s natural environment and cultural heritage, offer a valuable case for developing a strategy to distinguish purebred animals from crossbreds and to identify pedigree errors. Several cattle breeds are currently raised on the island. Among the five most common, two are local beef cattle breeds (Sardo Bruna and Sarda), one is a cosmopolitan and under-selection beef cattle breed (Limousine), and the remaining are largely crossbreds. The widespread, extensive, and often multi-breed rearing system complicates breed identification and accurate pedigree registration. Nevertheless, the current population of Sardo Bruna and Sarda could pave the way for removing these breeds from the national list of animals with limited diffusion (at risk of extinction). Should this occur, a reliable strategy will be necessary to quickly and accurately verify the breed of the animal, particularly for meat marketing, product valorization, or breed-specific selection programs. This study lays the groundwork for using an innovative approach by exploiting breeds specifically linked to selection signatures.

### Population structure analysis

The PCA accurately depicted the population structure of the four breeds, highlighting that the local breeds are genetically closer to the Limousine breed (Fig. 2). Indeed, it is known that Sarda, historically a beef breed, was crossed with Bruna and Modicana, both dairy cattle breeds, to improve milk production. As expected, it was positioned between the other two local breeds. These findings emphasize the need to develop an approach capable of distinguishing Sarda and its derivatives using a low-cost strategy, tailored to Sardinian cattle management systems. Furthermore, the PCA identified several potentially admixed individuals, indicating crossbreeding within the studied populations. In particular, 253 animals from the Sardinian breeds and 62 individuals recorded as Limousine showed intermediate positions between clusters, raising doubts about their breed status. These cases underscore the importance of systematic ancestry verification and the potential utility of genomic tools.

### Selection signature analyses

The iHS test was employed to detect recent selection signatures within the population. Subsequently, the XP-EHH approach was applied to identify selective sweeps differentiating populations, while the F_ST_ method was used to assess allele frequency divergence among breeds. The combination of these approaches enabled us to detect both shared and population-specific genomic regions potentially under selection. Forty-two genes were located in common selection signatures shared across all four beef breeds (Fig. 4). On the other hand, 6 and 24 genes were located in breed-specific selection signatures in SAB and SAR, respectively.

In SAB, a selection signature was detected in both iHS and XP-EHH in a region on BTA6 harboring the genes *LAP3*, *MED28*, *FAM184B*, *DCAF16*, *NCAPG*, and *LCORL*. These genes have been identified in several studies as candidate genes for body and bone weight, as well as growth and development [13–17]. In particular, *NCAPG* and *LCORL* form a well-known growth-related QTL complex, with polymorphisms repeatedly associated with traits such as average daily gain, carcass weight, and feed intake, as reported by Lindholm-Perry et al. (2011) [18] in Limousine and Charolais populations. In a genome-wide association study based on whole-genome sequence data, Niu et al. (2021) [17] identified *LAP3*, *MED28*, *NCAPG*, *LCORL*, and *FAM184B* as significantly associated with bone weight in Chinese Simmental cattle. Similarly, Baneh et al. 2025 [19] reported overlapping selection signals in the same ~ 38 Mb region on BTA6, encompassing *LAP3*, *MED28*, *DCAF16*, *NCAPG*, and *LCORL*, in three Iranian cattle breeds, using a combination of XP-EHH and F_ST_. Lindtke et al. (2024) [20] further confirmed this haplotype block by demonstrating extensive linkage disequilibrium in cattle beef crossbreds, reinforcing the hypothesis of long-term selection pressure in this genomic region.

Among the 24 genes related to the selection signatures specific for SAR, interesting were (i) the *RPS6KA1* which plays a key role in hepatic metabolic adaptation by inhibiting oxidative stress [21], (ii) *HMGN2*, which has been revealed as a potential candidate gene linked to the fore udder morphology due to its antimicrobial activity against bacteria, viruses, and fungi [22]; (iii) *ZNF683* found to contribute to mastitis development [22]; (iv) *UBXN11*, a gene belonging to the UBX family member proteins important to regulate diverse cellular processes, including protein stability and degradation [23]; (v) *CATSPER4* which plays an essential role in sperm hyperactivated motility and male fertility in cattle [24], while (vi) *FAM110D*, and *ZNF593*, both were identified in a genomic region associated with lactation persistency in Holstein cattle [25]. The *CATSPER4*, *CNKSR1*, *ZNF593*, and *FAM110D* genes described above were also found in heterozygosity hotspots in Italian beef breeds [26]. At the same time, they are identified in the genomic windows upstream or downstream of the significant iHS signals, revealing a marked increase in extended haplotype homozygosity. Lastly, *PDIK1L* has been found to regulate the Zn concentration in dairy milk [27]. Among the genes identified in SAR, several stand out for their functional relevance and alignment with previous findings that highlight the role of local adaptation and selection in shaping the bovine genome. The presence of *CATSPER4*, involved in sperm motility, and *FAM110D*, linked to lactation persistency, underscores the selective pressure on both reproductive efficiency and milk production, two traits of economic relevance in local breeds managed under low-input systems. Similar dual-purpose dynamics were observed in studies of Creole and African cattle, where selection signals overlapped with genes influencing fertility, lactation, and stress response [28, 29]. Moreover, the detection of *HMGN2* and *ZNF683*, both involved in immune responses, resonates with the growing body of evidence showing that immunity-related genes are often targeted by natural selection in local breeds exposed to environmental challenges. Although such patterns have been particularly well-characterized in goats [30], they are also increasingly reported in cattle, especially in rustic populations adapted to marginal territories. The co-localization of these genes with iHS peaks and heterozygosity hotspots in SAR supports the hypothesis of both recent and ancient selective events, shaping a genomic profile compatible with resilience, productive stability, and environmental adaptation. This pattern resembles what has been described in the Podolica breed, where selection signals have been associated with genes involved in lipid metabolism (e.g., *FASN*), cellular stress response (e.g., *DNAJC5B*), and innate immunity (e.g., *BOLA-DQA2*) [31]. While these genes differ from those identified in SAR, the overlap in functional categories, particularly those related to oxidative stress regulation (*RPS6KA1*), immune competence (*HMGN2*, *ZNF683*), and reproductive traits (*CATSPER4*), suggests convergent adaptive responses to similar environmental constraints in extensive and low-input production systems.

Regarding genomic regions associated with the top 0.1% of cross-population extended haplotype homozygosity (|XP-EHH|) scores, three significant segments were identified on BTA6, BTA11, and BTA14 (Table 4), which were also supported by F_ST_ values. In addition, a region on BTA 5 (from 48,229,383 − 48,333,093) was under positive selection in LIM, when compared with SAR. Here, *MSRB3* is located, a gene previously associated with growth and fat deposition traits [32], with indel polymorphisms influencing carcass composition.

Pairwise comparisons of extended homozygous haplotypes identified a region on BTA6 as a nearly fixed or ongoing signature of selection in SAB and SAM. On BTA11, haplotypes were specific to SAB, while the region on BTA14 distinguished the LIM breed from all the local populations.

Nine genes are allocated in the region on BTA6 from 36,681,743 to 38,001,622 bp (*SPP1*, *MEPE*, *IBSP*, *LAP3*, *MED28*, *FAM184B*, *DCAF16*, *NCAPG*, and *LCORL*). This region was also identified in SAB using the iHS approach and has been previously associated with many economically important traits in beef and dairy cattle, including QTLs related to carcass weight, average daily gain, body weight and conformation, calving ease, calving interval, milk yield, and composition (https://www.animalgenome.org/cgi-bin/QTLdb/BT/index). Six of the nine genes in the genomic region, namely *LAP3*, *MED28*, *FAM184B*, *DCAF16*, *NCAPG*, and *LCORL*, have been identified in several studies as candidate genes of body and bone weight, as well as growth development [13–17]. Matsumoto et al. (2019) [33] also identified the *SPP1* gene (secreted phosphoprotein 1) as an additional candidate gene for carcass traits, revealing a mutation that could improve beef quality. The last two genes of the previously described cluster, i.e., *IBSP* (Integrin Binding Sialoprotein) and *MEPE* (Matrix Extracellular Phosphoglycoprotein) genes, were identified as potential candidates for milk protein and milk fat percentage in Israeli Holstein [34] and for dystocia and stillbirth in Norwegian Red cattle [35], highlighting the region’s relevance also for fertility traits.

The consistent identification of selection signatures on BTA6, particularly encompassing *NCAPG*, *LCORL*, and *LAP3*, aligns with well-documented associations with growth and carcass traits in beef cattle. Majeres et al. (2024) [36], using whole-genome sequencing in Charolais-sired calves, refined a ~ 814 kb haplotype including these genes, which was significantly associated with birth weight, live weight, carcass weight, hip height, and average daily gain. Complementary evidence was also provided by Zhang et al. (2018) [37], who performed a comparative analysis across seven Chinese local breeds from distinct geographical regions. In northern populations, characterized by Simmental introgression, they observed strong signals of selection in the same genomic region, particularly highlighting *NCAPG*, *LCORL*, *LAP3*, and *MED28*, with associations related to meat yield and body size. Zhang et al. (2024) [38], in a study on Xinjiang Brown cattle, a dual-purpose breed well adapted to an arid environment, detected overlapping selection peaks on BTA6, reinforcing the relevance of this region for growth performance and metabolic regulation. Garduño-López et al. (2024) [39] also identified converging signals in Mexican zebu-influenced breeds, particularly in those subjected to selection for frame size and carcass traits, emphasizing the presence of *LAP3*, *MED28*, and *IBSP* within XP-EHH peaks. These genes are potentially involved in skeletal development and mineral metabolism.

Beyond these core loci, additional candidate genes reported in these studies, such as *MEPE*, *FAM184B*, and *IBSP*, may contribute to structural conformation and tissue mineralization processes. These findings suggest a degree of functional convergence despite the contrasting production environments and genetic backgrounds of the populations analyzed. The recurrence of signals in analogous genomic regions underscores the evolutionary and economic importance of this segment on BTA6 (36,681,743 − 38,001,622 bp) in shaping growth-related phenotypes under diverse selective pressures.

The region on BTA11 (from 66,502,254 to 69,619,152 bp) is rich in genes (*n* = 32) and harbors numerous QTLs associated with both milk composition and body weight. Indeed, this region has been previously identified by Signer-Hasler et al. (2023) [40] as a selection signature in several Alpine cattle breeds. Rothammer et al. (2013) [41] and Reding et al. (2023) [42] proposed seven of the 32 genes identified in the present study as candidates related to fertility traits (i.e., *PROKR1* (Prokineticin Receptor 1), *GFPT1* (Glutamine–Fructose-6-Phosphate Transaminase 1), *EHD3* (EH Domain Containing 3), *GMCL1* (Germ Cell-Less 1, Spermatogenesis Associated), and *PCBP1* (Poly(RC) Binding Protein 1) *- AAK1* (AP2 Associated Kinase 1) and *CNRIP1* (Cannabinoid Receptor Interacting Protein 1) genes, respectively). Other genes located in this region on BTA11, such as *CAPN14* (Calpain 14), *CAPN13* (Calpain 13), *LBH* (LBH Regulator Of WNT Signaling Pathway), and *LCLAT1* (Lysocardiolipin Acyltransferase 1), have been associated with meat quality traits [33, 34], while *GALNT14* (Polypeptide N-Acetylgalactosaminyltransferase 14) has been proposed as a candidate gene for milk composition traits [43]. These findings reinforce the SAB’s aptitude for milk production, as this selection signature was revealed exclusively in this breed.

Finally, the region on BTA14 (from 22,781,305 to 24,452,175 bp) harbored 11 genes, including *XKR4* (XK Related 4) and *TMEM68* (Transmembrane Protein 68), which are candidate genes for feed intake and growth development in crossbreed cattle [44]. Other notable genes in this region - *LYN (LYN Proto-Oncogene*,* Src Family Tyrosine Kinase)*, *RPS20* (Ribosomal Protein S20), *MOS* (MOS Proto-Oncogene, Serine/Threonine Kinase), *PLAG1* (PLAG1 Zinc Finger), *SDR16C5*, *SDR16C6* (Short Chain Dehydrogenase/Reductase Family 16 C Member 5 and 6, respectively) and *PENK* (Proenkephalin) - have been linked to feed efficiency and growth traits in U.S. beef cattle by Seabury et al. (2017) [45].

## Conclusion

This study represents the first investigation of selection signatures using the integrated haplotype score (iHS) statistics and cross-population extended haplotype homozygosity (XP-EHH) in the four cattle beef breeds mainly reared in Sardinia (Sarda, Sardo Bruna, Sardo Modicana, and Limousine).

These results provide the basis for elaborate valorization strategies that will be cheaper and useful for farmers’ and breeders’ associations. An essential outcome of this study is the identification of genomic regions and candidate genes repeatedly associated across breeds, which could serve as a core panel for differentiating purebred animals from potential crossbreds in geographically isolated contexts such as islands. Developing robust genomic strategies to accurately discriminate between pure and admixed individuals is crucial for preserving genetic diversity, especially in closed environments with limited gene flow. Our findings highlight specific loci, such as those on BTA6, BTA11, and BTA14, with consistent selection signatures and harbor genes associated with growth, fertility, and production traits, making them promising targets for traceability and breed authentication. Incorporating such genomic markers within breeding and conservation programs will enhance the accuracy of pedigree verification, minimize errors related to crossbreeding, and support sustainable management of local genetic resources.

## Supplementary Information

Additional file 1: The 42 genes located in the common selection signatures among the four cattle beef breeds.

Additional file 2: The top 0.01% XP-EHH values for each pairwise comparison.

Additional file 3: Figure S1.

Additional file 4: The genes located within the top 0.01% F_ST_ values and details compared to genes obtained by XP-EHH scores.

## Data Availability

The datasets generated and analysed during the current study are not publicly available because they are owned by the National Association of Breeders of Limousine and Charolaise breeds (ANACLI, Roma, Italy; email: anacli@anacli.it) but are available from the corresponding author on reasonable request.

## Abbreviations

BTA: Bos taurus autosome
EHH: Extended haplotype homozygosity
IHH: integrated haplotype homozygosity
iHS: Integrated haplotype score
LIM: Limousine
LD: Linkage disequilibrium
MAF: Minor Allele Frequency
PCA: Principal Components Analysis
rEHH: relative extended haplotype homozygosity
ROH: Runs of Homozygosity
SAB: Sardo Bruna
SAM: Sarda Modicana
SAR: Sarda
SNP: Single Nucleotide Polymorphism
XP-EHH: cross-population extended haplotype homozygosity
