# Supplementary material for: Key genomic regions identified through selection signatures distinguish cattle breeds reared in Sardinia Island
Source: BMC Genomics. 2025 Nov 5;26:995. doi: 10.1186/s12864-025-12204-6 (PMC12587522; doi:10.1186/s12864-025-12204-6)

**Supplemetary figures**

Figure S1: Heatmap of pairwise SNPs Linkage Disequilibrium for each significant homozygous haplotype (A – on BTA6; B – on BTA11; C – on BTA14).

A)


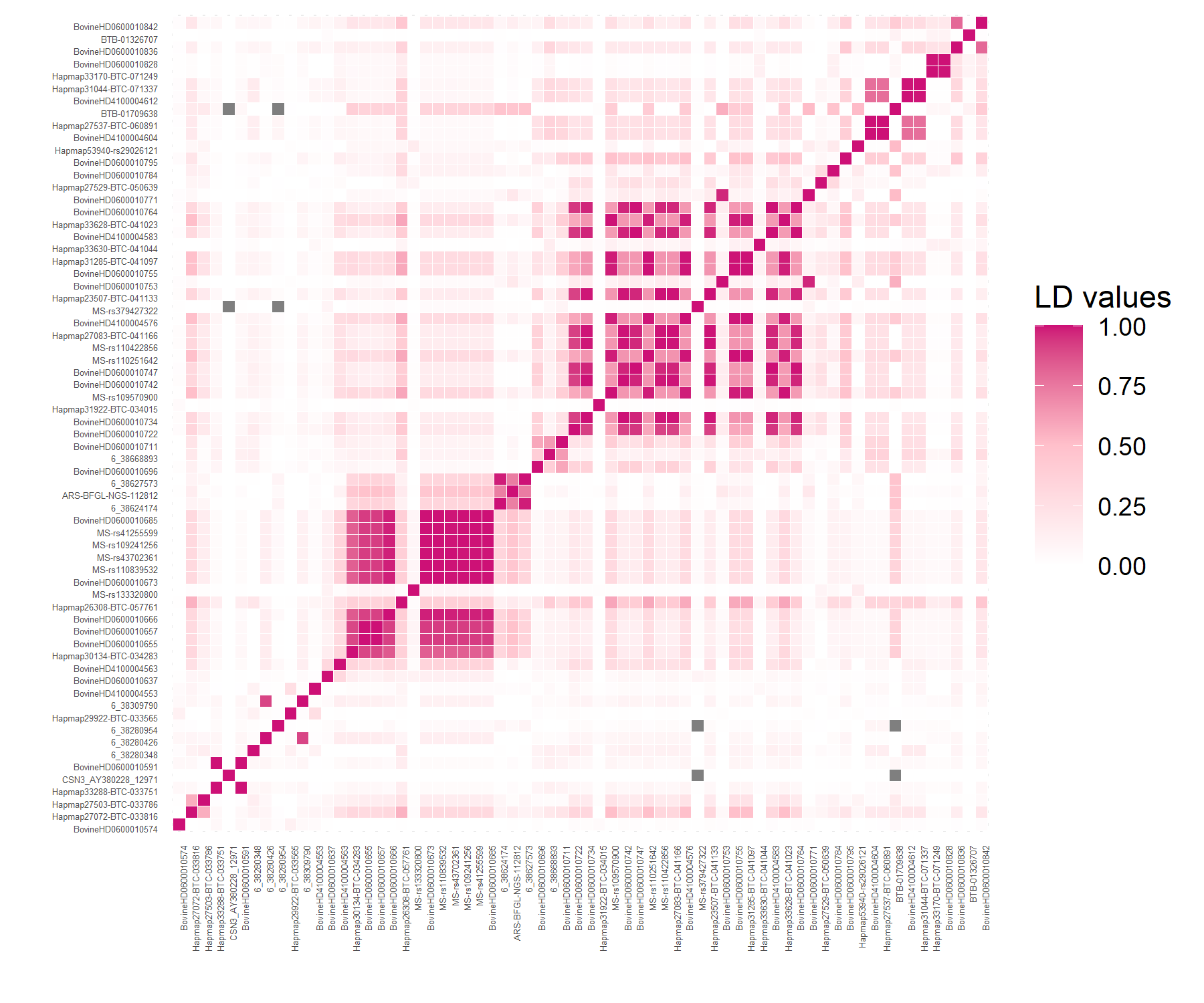


B)


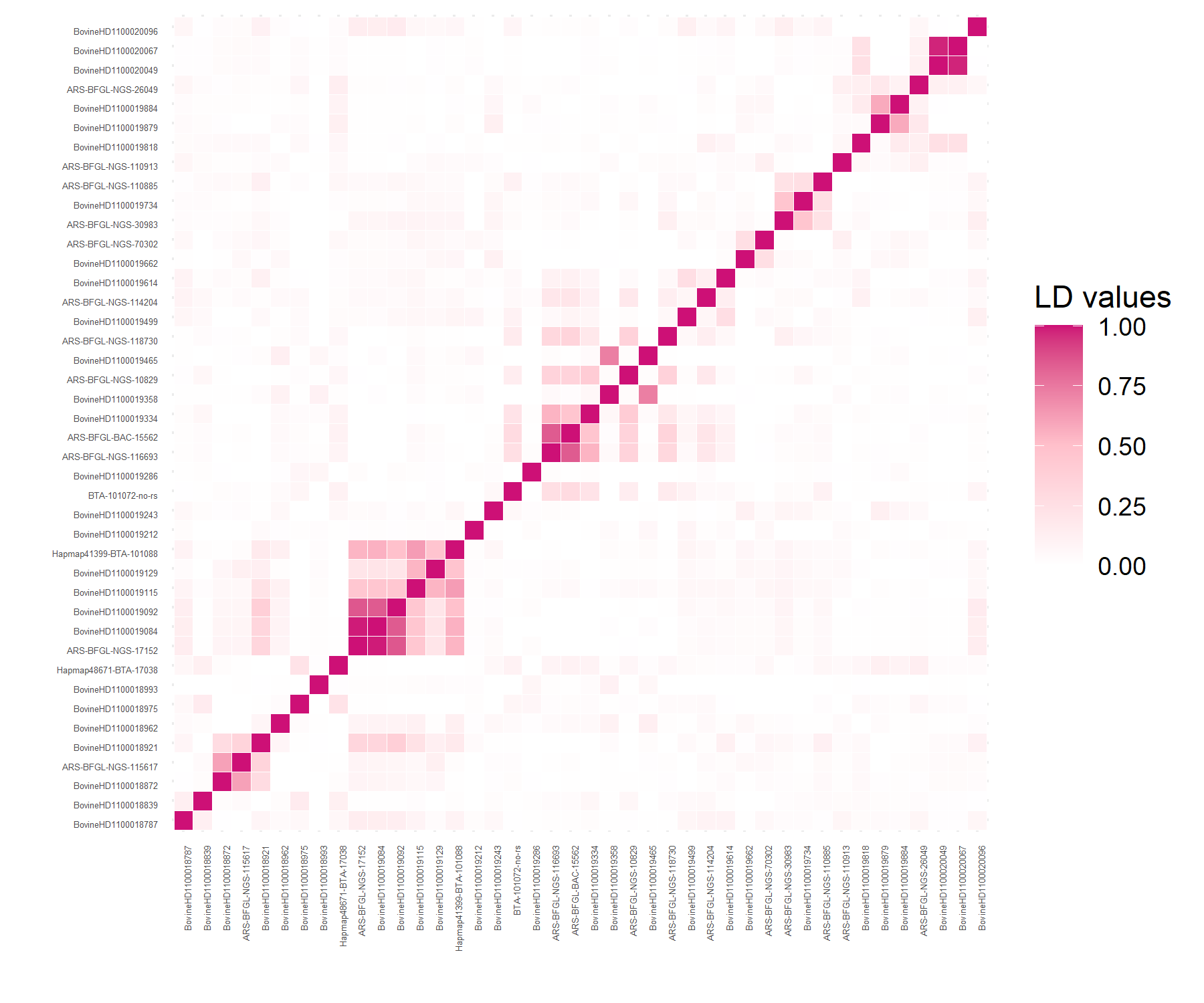


C)


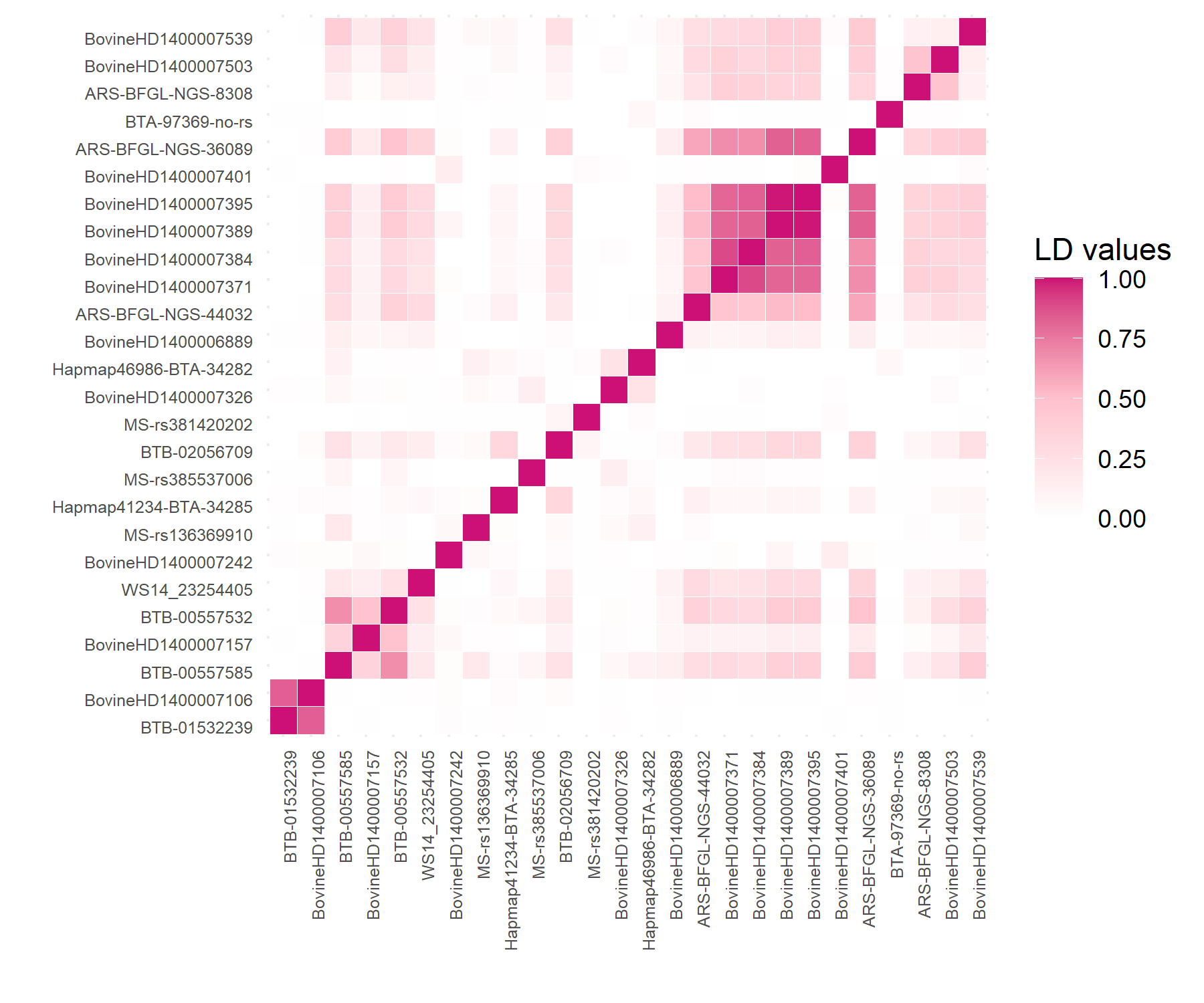

Supplement: Supplementary file 3 — Additional file 3: Figure S1. [file 12864_2025_12204_MOESM3_ESM.docx]
